# The Effect of the Treatment at a Pain Clinic on the Patients’ Assessment of Their Pain Intensity and the Incidence of Mental Disorders in the form of Anxiety, Depression, and Aggression

**DOI:** 10.3390/ijerph16040586

**Published:** 2019-02-18

**Authors:** Dariusz Kosson, Marcin Kołacz, Robert Gałązkowski, Patryk Rzońca, Barbara Lisowska

**Affiliations:** 1 Department of Anaesthesiology and Intensive Care, Division of Teaching, Medical University of Warsaw, 4 Lindley St., 02-005 Warsaw, Poland; kosson@wp.pl; 2 Department of Anaesthesiology and Intensive Care, Medical University of Warsaw, 4 Lindley St., 02-005 Warsaw, Poland; mkolacz66@gmail.com; 3 Department of Emergency Medical Services, Faculty of Health Science, Medical University of Warsaw, 81 Żwirki i Wigury St., 02-091 Warsaw, Poland; r.galazkowski@lpr.com.pl; 4 Department of Emergency Medicine, Faculty of Health Sciences, Medical University of Lublin, 4-6 Staszica St., 20-081 Lublin, Poland; patryk.rzonca@gmail.com; 5 Department of Anesthesiology and Intensive Care, John Paul II Western Hospital in Grodzisk Mazowiecki, 11 Daleka St., 05-825 Grodzisk Mazowiecki, Poland

**Keywords:** pain, anxiety, depression, aggression, pain clinic, pain intensity, treatment effectiveness

## Abstract

The aim of the study was to analyze the effect of the treatment given to patients in a pain clinic on their assessment of pain intensity and the incidence of emotional disturbances in the form of anxiety, depression, and aggression. The study was conducted from January 2014 to April 2018 among patients under the care of two Warsaw pain clinics. The study tools were the Hospital Anxiety and Depression Scale—Modified Version (HADS-M) and the Numerical Rating Scale (NRS). The project enrolled 325 patients, with women comprising 60.62% of patients, and the age bracked of 65–79 years comprising 39.38% of patient. The major reasons for attending the pain clinic were osteoarticular pain (44.92%) and neuropathic pain (42.77%). The therapy applied lowered the patients’ pain intensity (4.98 vs. 3.83), anxiety (8.71 vs. 8.12), aggression (3.30 vs. 3.08), and the overall HADS-M score (18.93 vs. 17.90), which shows that the treatment of both the pain symptoms and the associated emotional disturbances in the form of anxiety and aggression was effective. Sex is a factor affecting pain intensity. The level of mental disorders was influenced by the sex and age of the patients and how long they had been treated in the pain clinics.

## 1. Introduction

According to the International Association for the Study of Pain (IASP), which promotes research in the scientific, practical, and educational aspects of this field [1,2], pain is defined as “an unpleasant sensory and emotional experience associated with actual or potential tissue damage or described in terms of such damage” [2] (p. 5). For each individual it is a unique sensory experience, characterized by location, intensity, duration, and type. Chronic pain is one of the most frequent reasons why adults seek help from medical professionals. It is connected with restricted mobility, frequently overusing analgesics to perform daily tasks, experiencing anxiety and depression, a poor assessment of one’s health, and lowering one’s quality of life [3,4,5]. The estimated data regarding adult Americans point out that chronic pain affects from 11% to 40% of the population. In 2016, there were 20.4% (50 million) adult Americans who suffered from chronic pain, and 8.0% (19.6 million) who experienced chronic pain of great intensity [6].

Chronic pain is a little-known condition which poses a major challenge to modern medicine, since it is difficult to diagnose and to find appropriate management for each case. In spite of the continuous development of medicine resulting in greater accessibility of new treatment methods and the development of new-generation analgesics, many attempts to eliminate pain from everyday life end in failure. The International Association for the Study of Pain (IASP) recommends interdisciplinary treatment of patients with chronic pain, underlining the need to address not only the aspect of the patients’ somatic feelings but also biological, psychological, and social factors in the model of pain management and to implement psychological, social, recreational, and professional training. Pharmacotherapy remains the basic method of treatment, but it should comprise an element of a complex program of rehabilitation, whose final effect is not only to lower pain intensity but also improve the patients’ quality of life and make it possible for them to return to their families and to society [7,8,9,10].

Since the process of diagnosing and treating pain is complex, it requires comprehensive planning, which demands specialist knowledge and experience from doctors dealing with the problem of pain management. Interdisciplinary treatment of patients suffering from pain is possible in specialized organizational units that are being set up in many countries. An example of such units are clinics and counseling centers for pain management. Their primary objectives are to treat patients with acute and chronic pain, educate both healthcare personnel and patients, and participate in the preparation of guidelines and research in the field of pain [10,11,12].

The problem of pain management has now become such an important issue that the authors decided to undertake research aiming to analyze the effect of the treatment offered in pain clinics on the intensity of pain and the incidence of emotional disorders in the form of anxiety, depression, and aggression.

## 2. Materials and Method

### 2.1. Participants

Research was conducted in patients under the care of two outpatient pain clinics in the city of Warsaw: The Pain, Anesthesiology and Intensive Therapy Clinic of the Medical University of Warsaw, 4 Lindleya Street, as well as the Pain Clinic located at 231 Czerniakowska Street (Poland). It took place from January 2014 to April 2018. The inclusion criteria for participation in the study were as follows: 18 years of age, ascertainment of pain on the basis of the patient’s medical history, and attending a pain clinic in the city of Warsaw. The exclusion criterion was the lack of informed consent on the part of the patient. The study obtained approval no. AKBE/24/15 from the Bioethics Committee of the Medical University of Warsaw. It was, moreover, approved by the managers of the clinics involved. The patients taking part in the project were informed about the voluntary nature of their participation, their anonymity, and the fact that the results of the study would be used exclusively for scientific purposes. The study assessed the level of pain intensity and emotional disturbances in the form of anxiety, depression, and aggression in the patients who agreed to participate in it during their first and subsequent visit in the above-mentioned pain clinics.

### 2.2. Assessment

Research was based on the analysis of the medical histories of the patients attending the pain clinic (their sociodemographic data) as well as by the diagnostic survey method using the questionnaire technique. The research tools were: The Hospital Anxiety and Depression Scale—Modified Version (HADS-M) and the Numerical Rating Scale (NRS).

The Hospital Anxiety and Depression Scale—Modified Version (HADS-M) is a revision of the Hospital Anxiety and Depression Scale (HADS) developed by Zigmond and Snaith [7], and was adapted for Polish conditions by Majkowicz, de Walden-Gałuszko, and Chojnacka-Szawłowska. HADS-M comprises three subscales to assess anxiety, depression, and aggression. The subscale of both anxiety and depression consists of seven statements, while the subscale of aggression has two. Answers are given on a scale of 0 to 3 points. The maximum score for the anxiety and depression subscale is 21, while for the aggression subscale it is 6 points. Higher results on each subscale point to greater disturbances. Anxiety, depression, and aggression are assessed and interpreted separately using the percentage reference to the score (lack of disturbances: 0–33.33%; borderline cases: 33.34–47.62%; the occurrence of threat: 47.63–100%) or using the score matrix: lack of disturbances: 0–7 points for anxiety and depression, 0–2 points for aggression; borderline cases: 8–10 points for anxiety and depression, 3 points for aggression; disturbances occurring: 11–21 points for anxiety and depression, 4–6 points for aggression [13,14,15]. 

The Numerical Rating Scale (NRS) is a one-dimensional measurement of pain intensity in adults. The most frequently used version is the 11-point NRS scale, which was the one used in the present study. The NRS form can be filled out by the patients either by reporting the scores verbally or by writing them down on their own. The respondents are asked to rate their pain giving a numerical score to its intensity. The NRS score ranges from 0 to 10, with 0 indicating no pain and 10 the worst possible pain. Higher scores on the NRS scale indicate a higher intensity of pain [16,17].

### 2.3. Statistical Analysis

The data collected from the survey questionnaires were analyzed using the STATISTICA version 13 software (StatSoft, Kraków, Poland). Amounts and percentages were used in the description of quality data, while in numerical data—the mean (*M*) and standard deviation (*SD*) are shown.

The normality of variable distribution was performed using the Shapiro-Wilk normality test. The two dependent samples (on the one hand the intensity of pain and on the other the level of anxiety, depression, and aggression) were compared using the Wilcoxon matched-pairs signed-ranks test. The differences between the two groups were analyzed by means of the Mann-Whitney U-nonparametric test, while the Kruskall-Wallis test was performed to compare more than two groups of variables. The study assumed statistical significance as *p* < 0.05.

## 3. Results

The study enrolled 325 patients treated in pain clinics. Women dominated in this group (they comprised 60.62% of those enrolled), while most of the overall group of patients were in the 65–70 age bracket (39.38%), with the mean age being 62.17 years (*SD* 14.73). The main reasons for attending the pain clinic were osteoarticular pain (44.92%) and neuropathic pain (42.77%). Over half of the group studied were given specialist multimodal therapy treatment (59.69%), with antidepressant drugs being the most frequent medication (56.31%). The patients had usually been attending a pain clinic for over two years (29.54%) (Table 1).

The statistical analysis that was conducted—which took into account both the changes in pain intensity measured on the NRS scale and emotional disturbances in the form of anxiety, depression, and aggression evaluated on the HADS-M scale between the first and the subsequent visit in the pain clinic—showed a statistically significant difference in the case of pain intensity, the level of anxiety and aggression, and the overall result on the scale. As a result of the therapy undertaken in the pain clinics, the patients obtained lower values expressing pain intensity, anxiety, depression, and the overall HADS-M score. In the case of depression, the difference was not statistically significant (Table 2).

Analysis showed statistically significant differences during the first visit in pain clinics between the sex and age of patients compared to the level of anxiety, and, similarly, between the duration of treatment versus the level of depression as well as between the duration of treatment compared to the level of aggression (*p* < 0.05). It follows from the study that women and younger people had a higher level of anxiety during the first visit, patients who had been treated for 4 to 12 months showed the highest level of depression, and the group of patients under 34 years of age who had been treated at the pain clinic for between four and twelve months showed a significantly higher level of aggression. When examined on the subsequent visit, the analysis indicated significant differences between pain intensity versus the level of anxiety, the duration of treatment versus the level of depression, and the age of the patients versus the level of aggression (*p* < 0.05). During the subsequent visit at the pain clinic, women reported a significantly higher pain intensity and anxiety level than men, and patients aged 35–49 demonstrated the highest level of aggression. A significantly higher level of depression was found in patients who had been treated for two years or longer. Table 3, Table 4 and Table 5 show the detailed statistical analysis.

## 4. Discussion

For each individual, pain is a unique, unpleasant sensory experience, which is connected with a limitation of mobility, reduced ability to perform daily tasks, a decreased quality of life, and poor assessment of health. It is perceived by patients as a disease and suffering, which in the long run can give rise to emotional problems that have a negative impact on themselves, their family, and those who are close to them, and moreover has social consequences [1,2,18].

This is why the authors of the present paper undertook the analysis of the impact that pain intensity and the occurrence of emotional disturbances in the form of anxiety, depression, and aggression has on the life of the patients attending pain clinics.

Women dominated in the analyzed group of patients, as well as people aged from 65–79, with the mean age being 62.17 years. Similar demographic data were investigated in the study of McLoughlin et al. (2018) devoted to adult patients treated in the University Hospital Limerick in Ireland [19]. Moreover, women also constituted the majority of patients in the study of Tiippan et al. (2016), which researched the factors of chronic post-operational pain among the patients with acute pain treated at the Helsinki University Hospital, where the mean age was 46 years [20]. The results of our own study showed that the most frequent reasons for using the help of a pain clinic were osteoarticular pain and neuropathic pain. The duration of treatment was usually over two years, and over half of the patients were given specialist multimodal treatment. It follows from the study of Batisaki et al. (2018) done in patients at the pain ward of the Attikon University General Hospital in Athens that the type of pain reported most frequently was located in the area of the lower back (sciatica) and headache [21]. Rockett et al. (2013) did research among the patients of a pain management center in Plymouth that indicated that patients most often presented with back pain and secondly musculoskeletal and neuropathic pain [22]. The analysis of Brzeziński et al. (2009) reveals that back pain and headache were the most frequent reasons why patients saw doctors at the pain clinic of the Institute of Rural Health in Lublin [23].

In the present study, the authors made two measurements to assess the pain intensity and the level of emotional disturbances. The first one was performed in the pain clinic when the patients first came there and the next one was performed during their subsequent visit. It follows from the analysis conducted that the level of pain intensity, anxiety, aggression, and the overall result on the HADS-M scale underwent significant changes as a result of treatment in the pain clinics: the respective scores fell and were 4.98 vs. 3.83 for pain intensity, 8.71 vs. 8.12 for anxiety, 3.30 vs. 3.08 for aggression, and 18.93 vs. 17.90 for the overall HADS-M. Thus, both the treatment of pain intensity given at the pain clinic and the associated emotional disturbances in the form of anxiety and aggression proved to have been effective. Pain is closely linked to the occurrence of emotional disturbances, which is confirmed by the studies of Pakpou et al. (2018), Sagheer et al. (2013), and Ahmed et al. (2013) [24,25,26]. The study of Merrick et al. (2012), who did a year-long observation of two rehabilitation management strategies of chronic pain in patients treated at the rehabilitation clinic of the University Hospital of Umeå, Sweden, shows that the patients enrolled in the multimodal rehabilitation program reported a lack of pain symptoms in a statistically significant number of cases (59% vs. 46%). Moreover, as a result of rehabilitation, there was a fall both in their level of pain (7.2 vs. 6.4) and depression (8.0 vs. 5.4) [27]. The studies of Wong et al. (2011) comparing the frequency of mental disorders among the Chinese patients attending specialist orthopedic clinics and those treated at interdisciplinary pain treatment clinics showed that mental disorders in the form of depression and anxiety disorders occurred three to four times more often among pain clinic patients. As the authors indicate, the higher ratio of mental disorders among the patients of the pain clinic was due to the character of the pain and pain intensity. The patients attending the pain clinics presented with more pain locations, had suffered for a longer time, and experienced greater pain intensity, which influenced their decision to choose a specialist pain management center [28]. The study of Vanhaudenhuyse et al. (2014) assessed the effect of psychotherapy, therapeutic education, self-care, and self-hypnosis on the level of pain, the mental disturbances, and the quality of life among patients with chronic pain treated at the Department of Algology-Palliative Care Department, Belgium. The results of their research showed that a significant reduction of anxiety was observed as a result of using physiotherapy and psychoeducation as well as self-care and self-hypnosis. Depression subsided if self-care and self-hypnosis were implemented and pain intensity was reduced only as a result of implementing self-care and self-hypnosis [29]. The study of Martín et al. (2014), who did research in a group of patients with fibromyalgia referred to the pain management unit at the clinic of Hospital Galdakao-Usansolo, Spain, shows that using multimodal therapy leads to the reduction of disability resulting from pain symptoms, and reduces the level of pain, tiredness, and anxiety [30]. The results of the study were also confirmed in the research of Goldenberg et al. (2004), Mannerkorpi et al. (2009), King et al. (2002), and Dunkl et al. (2000) [31,32,33,34]. It is worth underlining that our own research results concerning emotional disturbances connected with pain were broadened by the aspect of aggression, which is a subscale in the HADS-M scale (i.e., a modification of the HADS scale adapted for Polish conditions) [14,16].

The further part of our study was devoted to assessing the impact of sex, age, and duration of treatment on the level of pain intensity and disorders taking the form of anxiety, depression, and aggression. The results of our own studies indicated that during the first visit women scored higher on the scale of anxiety, while during the subsequent visit they scored higher on both anxiety and pain intensity in comparison to the studied group of men. Patients in the 35–39-year age bracket demonstrated the highest level of anxiety, while those under the age of 34 years had the highest level of aggression both during the first and the subsequent visit. Patients whose duration of treatment was from 4 to 12 months indicated the highest levels of depression and aggression during their first visit, while during the subsequent visit those who had been treated for over two years demonstrated the highest level of depression on the HADS-M scale. In the study done by Vanhaudenhuyse et al. (2014), the authors also pointed out the influence of sex on patients with chronic pain. They observed that women indicated a higher level of anxiety and depression measured on the HADS scale. Similar results were reported in the study by Nolen-Hoeksema (2001) [29,35]. It emerges from the cross-sectional study on neuropathic knee pain and associated risk factors done by Fernandes et al. (2018) in Great Britain that this kind of pain more often affected younger women, particularly ones with a higher BMI, hypertension, hyperlipidemia, and diabetes, those who had suffered a knee injury, individuals who do high-risk jobs, and women who suffer from generalized pain disorders and have higher scores for anxiety and depression [36].

The authors of the present paper undertook this study in order to report the influence of the treatment of patients in pain clinics because they found no such reports in Polish literature. The study has some limitations. It was done in only two pain clinics in Poland and reports only a few types of pain. Moreover, the authors analyze selected determinants. However, due to the importance of pain management and associated mental disorders in the world today, it is reasonable to continue research on this subject. A better understanding of this field of knowledge will improve the social awareness of the existence of pain clinics and help ensure the best possible diagnostics and management as well as the high quality of specialist care in pain clinics.

## 5. Conclusions

The most frequent reason for patients undertaking treatment in pain clinics was osteoarticular and neuropathic pain. Women and people in the 65–79 age bracket were the most frequent patients in this group. Treatment in pain clinics has a positive impact on pain intensity and the level of anxiety and aggression in patients with pain symptoms. Sex is a factor that influences the level of pain intensity. The level of mental disorders is influenced by the patients’ sex and age.

## Figures and Tables

**Table 1:** Characteristics of the patients examined.

	*n* (%)
**Sex**	
Female	197 (60.62)
Male	128 (39.38)
**Age (%)**	
<34 years	19 (5.85)
35–49 years	39 (12.00)
50–64 years	112 (34.46)
65–79 years	128 (39.38)
≥80 years	27 (8.31)
Age (years)–mean (*SD*)	62.17 (14.73)
**Type of pain**	
Osteoarticular	146 (44.92)
Neuropathic	139 (42.77)
Headache	43 (13.23)
Others	21 (6.46)
**Pharmacological treatment**	
Yes	194 (59.69)
No	131 (40.31)
**Medication**	
Antidepressants *	183 (56.31)
Benzodiazepines **	10 (3.08)
Sleeping pills ***	19 (5.85)
**Time of treatment in the pain clinic**	
Up to 3 months	94 (28.92)
4–12 months	68 (20.92)
13–23 months	67 (20.62)
2 years or more	96 (29.54)

* Amitriptyline, doxepine, citalopram, escitalopram, fluvoxamine, sertraline, venlafaxine, duloxetine, or trazodone. ** Diazepam, alprazolam, estazolam, or clonazepam. *** Zolpidem or zopiclone.

**Table 2:** Analysis of the effectiveness of treatment in pain clinics.

Variables	First Visit	Subsequent Visit	*p*-Value
**NRS***M*(*SD*)	4.98 (2.83)	3.83 (2.76)	0.0000
**HADS-M Anxiety***M*(*SD*)	8.71 (3.88)	8.12 (3.90)	0.0006
**HADS-M Depression***M*(*SD*)	6.92 (3.76)	6.70 (3.70)	0.1965
**HADS-M Aggression***M*(*SD*)	3.30 (1.65)	3.08 (1.78)	0.0129
**HADS-M Overall***M*(*SD*)	18.93 (7.64)	17.90 (7.67)	0.0027

NRS: Numerical Rating Scale; HADS-M: Hospital Anxiety and Depression Scale—Modified Version.

**Table 3:** Statistical analysis of pain intensity and emotional disturbances during the first and subsequent visit at the pain clinic depending on the sex of the patients examined.

Variables	First Visit				Subsequent Visit			
	NRS *M*(*SD*)	HADS-M Anxiety *M*(*SD*)	HADS-M Depression *M*(*SD*)	HADS-M Aggression *M*(*SD*)	NRS *M*(*SD*)	HADS-M Anxiety *M*(*SD*)	HADS-M Depression *M*(*SD*)	HADS-M Aggression *M*(*SD*)
**Sex**								
Female	5.14 (2.89)	9.27 (3.77)	7.07 (3.75)	3.28 (1.64)	4.08 (2.77)	8.62 (3.79)	6.93 (3.71)	3.01 (1.78)
Male	4.73 (2.74)	7.86 (3.90)	6.68 (3.80)	3.34 (1.68)	3.40 (2.54)	7.34 (3.96)	6.35 (3.67)	3.20 (1.78)
***p*-value**	0.2101	0.0004	0.3472	0.8736	0.0393	0.0020	0.1698	0.3547

**Table 4:** Statistical analysis of pain intensity and emotional disturbances during the first and subsequent visit at the pain clinic depending on the age of the patients examined.

Variables	First Visit				Subsequent Visit			
	NRS *M*(*SD*)	HADS-M Anxiety *M*(*SD*)	HADS-M Depression *M*(*SD*)	HADS-M Aggression *M*(*SD*)	NRS *M*(*SD*)	HADS-M Anxiety *M*(*SD*)	HADS-M Depression *M*(*SD*)	HADS-M Aggression *M*(*SD*)
**Age**								
<34 years	5.53 (2.14)	9.42 (3.61)	5.89 (3.03)	4.84 (1.50)	4.16 (2.65)	8.95 (4.20)	5.58 (3.83)	3.79 (1.87)
35–49 years	3.90 (2.89)	9.67 (4.30)	6.74 (3.82)	4.00 (1.50)	3.13 (2.54)	9.21 (4.04)	7.15 (3.24)	3.87 (1.36)
50–64 years	4.97 (2.68)	9.53 (3.72)	7.73 (4.10)	3.38 (1.65)	4.20 (2.73)	8.33 (3.84)	7.27 (4.28)	3.29 (1.85)
65–79 years	5.11 (3.04)	8.05 (3.72)	6.41 (3.31)	2.93 (1.52)	3.71 (2.84)	7.77 (3.83)	6.21 (3.19)	2.69 (1.64)
≥80 years	5.59 (2.48)	6.63 (3.63)	6.93 (4.31)	2.63 (1.64)	3.70 (2.84)	6.74 (3.80)	6.81 (3.63)	2.48 (2.01)
***p*-value**	0.1255	0.0023	0.1017	0.0000	0.2680	0.0994	0.2081	0.0004

**Table 5:** Statistical analysis of pain intensity and emotional disturbances during the first and subsequent visit at the pain clinic depending on the duration of treatment of the patients examined.

Variables	First Visit				Subsequent Visit			
	NRS *M*(*SD*)	HADS-M Anxiety *M*(*SD*)	HADS-M Depression *M*(*SD*)	HADS-M Aggression *M*(*SD*)	NRS *M*(*SD*)	HADS-M Anxiety *M*(*SD*)	HADS-M Depression *M*(*SD*)	HADS-M Aggression *M*(*SD*)
**Duration of treatment**								
up to 3 months	4.78 (2.76)	8.30 (4.37)	6.15 (4.13)	3.01 (1.60)	4.07 (2.78)	8.48 (4.58)	6.39 (4.18)	2.93 (1.72)
4–12 months	5.68 (2.57)	9.63 (3.16)	8.06 (3.27)	3.82 (1.74)	3.65 (2.94)	8.15 (3.88)	7.26 (3.15)	3.62 (1.85)
13–23 months	4.70 (2.93)	8.54 (3.65)	6.07 (3.28)	3.21 (1.52)	4.03 (2.60)	7.64 (3.08)	5.69 (3.12)	2.90 (1.72)
2 years and longer	4.89 (2.97)	8.59 (3.93)	7.45 (3.78)	3.28 (1.67)	3.59 (2.74)	8.08 (3.74)	7.31 (3.80)	2.99 (1.79)
***p*-value**	0.1499	0.1039	0.0003	0.0255	0.4655	0.7237	0.0120	0.0631
